# Effectiveness of continuous allergenic food intake for acute food protein–induced enterocolitis syndrome

**DOI:** 10.1016/j.jacig.2024.100232

**Published:** 2024-02-20

**Authors:** Masaaki Hamada, Yoshihiko Sakurai, Ichiro Tanaka

**Affiliations:** a Department of Pediatrics, Yao Municipal Hospital, Osaka, Japan; b Department of Legal Medicine, Nara Medical University School of Medicine, Kashihara, Japan

**Keywords:** Food protein–induced enterocolitis syndrome, FPIES, continuous allergenic food intake, CAFI, non–IgE-mediated food allergy, treatment

## Abstract

Continuous intake of allergenic food is a safe and efficient treatment strategy for patients with a prolonged course of acute food protein–induced enterocolitis syndrome. The initial dose, dose escalation rate, and starting age for continuous allergenic food intake need further clarification.

Food protein–induced enterocolitis syndrome (FPIES) is a non–IgE-mediated food allergy characterized by delayed and potentially severe gastrointestinal symptoms.^1^

Patients with acute FPIES with difficulty achieving tolerance require continuous allergen avoidance,^1^ with only a few other treatment options available.^2^^,^^3^ In this single-center hospital-based case series, we evaluated the effectiveness of continuous allergenic food intake (CAFI) in these patients.

This case series was approved by the ethics committee of Yao Municipal Hospital (approval no. YMH-012221-108 and YMH-031022-167). Written informed consent was obtained from all of the patients’ parents.

Patients meeting the diagnostic criteria for acute FPIES^1^ and with onset before age 3 years were enrolled from 2015 to 2021. Patients with levels of specific IgE antibody to allergenic foods greater than 0.35 IUA/mL were included and classified as having atypical FPIES. As a baseline survey before the initial oral food challenge (OFC), patients’ background information (eg, history of asymptomatic ingestion) was analyzed (see Table E1 in the Online Repository at www.jaci-global.org).

OFC using a single dose was performed under direct medical supervision. To prevent misdiagnosing atypical FPIES as IgE-mediated food allergy, only symptoms of atypical FPIES that occurred 3 or more hours after food intake were evaluated.

Patients with induced symptoms at any OFC were presented with the alternatives of either continuous avoidance or CAFI, depending on the severity of their FPIES symptoms. There were 2 CAFI methods, with the selection criteria for the “low-dose” (LD) group” and “extremely low-dose” (ELD) group as follows: the age at induction was not set; the LD group consisted of patients with mild symptoms (a single episode of vomiting and/or diarrhea at any OFC); the ELD group consisted of patients with repetitive vomiting at the second or third OFC. The CAFI starting dose was less than one-fifth of the OFC dose for the LD group and less than 1/100 of the OFC dose for the ELD group.

Both groups started CAFI at home almost daily. If no FPIES symptoms developed during CAFI, the dose was gradually increased as follows: initially, to 2, 3, 5, and 10 times the initial dose, and then up to 10 or 100 times after 1 month if the food was still tolerated (see Table E2 in the Online Repository at www.jaci-global.org).

We evaluated the desensitization rate in the patients who received CAFI. In this report, tolerance was defined as improving during the natural course of FPIES and desensitization was defined as improving with daily ingestion of allergenic food.

Data are expressed as means and SDs. All statistical analyses were performed using EZR.

This case series comprised 21 patients who underwent a maximum of 3 OFC sessions. Of the 21 patients, 15 (71.4%) had a history of asymptomatic ingestion. Eventually, 12 patients achieved tolerance, and 7 of 8 patients who underwent CAFI (4 in the LD group and 4 in the ELD group) achieved desensitization. The median age for starting CAFI was 33 months (interquartile range 18-44) (Fig 1).

**Fig 1:**
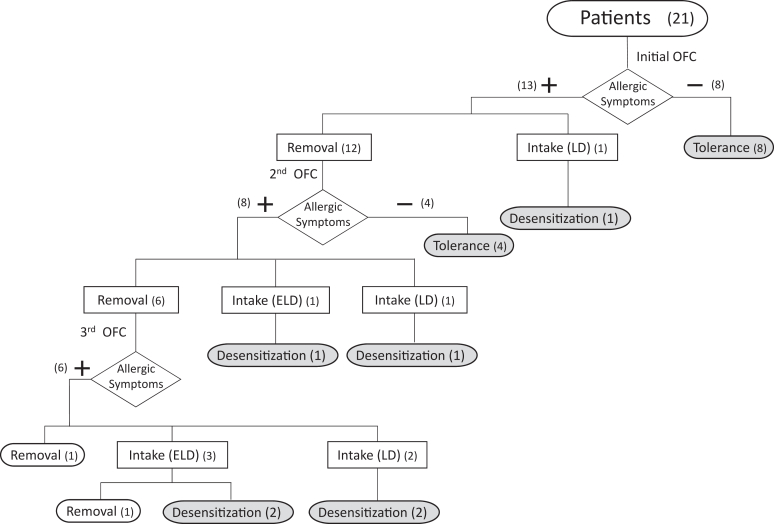
Study flowchart showing the patient distribution based on OFC results. During OFC, those patients who developed mild FPIES symptoms during OFC were assigned to the LD group, whereas those patients who experienced repetitive vomiting were assigned to ELD group. Numbers in parentheses indicate the number of patients.

In the LD group, all patients reached the dose triggering FPIES development within 1 month after starting CAFI and achieved desensitization within 3 months (Table I). One patient with wheat allergy complained of abdominal pain but subsequently achieved desensitization with CAFI. The other 3 patients achieved desensitization without developing FPIES symptoms during CAFI.

**Table I:** Characteristics of patients in the LD and ELD groups

Characteristic		Patient No.							
		1	2	3	4	5	6	7	8
Group		LD	LD	LD	LD	ELD	ELD	ELD	ELD
Allergenic food		Egg yolk	Wheat	Soy	Egg yolk	Milk	Egg yolk	Egg yolk	Wheat
Continuous intake starting age (mo)		17	55	11	27	38	49	42	18
Sex		Male	Male	Male	Female	Male	Female	Female	Female
Age at onset (mo)		6	5	8	7	12	8	11	8
Age at initial OFC (mo)		8	22	11	12	13	19	16	12
Age at second OFC (mo)		17	38		20	25	26	29	18
Age at third OFC (mo)			55		27	38	49	42	
Total IgE titer (IU/mL)		36	36	18	10	341	106	36	23
Specific IgE antibody titer to allergic foods (IUA/mL)		0.29	<0.10	<0.10	<0.10	0.42	42.9	6.64	<0.10
OFC dose and symptoms before the start of continuous intake	Allergenic food	Boiled egg yolk	Boiled udon	Soy milk	Boiled egg yolk	Milk	Boiled egg yolk	Boiled egg yolk	Boiled udon
	Dose	2 g	50 g	5 mL	2 g	2 mL	0.5 g	2 g	2 g
	FPIES symptoms	Single episode of vomiting	Single episode of vomiting	Single episode of vomiting	Single episode of vomiting	Frequent vomiting	Frequent vomiting	Frequent vomiting	Frequent vomiting
			Abdominal pain	Lack of activity		Abdominal pain	Lethargy	Lethargy	Abdominal pain
						Lethargy	Pallor	Pallor	Lethargy
Chronologic progression of continuous intake∗	Allergenic food	Boiled egg yolk	Boiled udon	Soy milk	Boiled egg yolk	Milk	Boiled egg yolk	Boiled egg yolk	Boiled udon
	Starting dose	0.2 g	10 g	1 mL	0.2 g	0.01 mL	0.01 g	0.01 g	0.04 mg
	Dose at 1 mo	2 g	50 g	35 mL	2 g	1 mL	0.1 g	Discontinuation	4 mg
	Dose at 2 mos	5 g	100 g	50 mL	5 g	5 mL	1 g		15 g
	Dose at the end of CAFI†	20 g	——	100 mL	20 g	100 mL	20 g		100 g
Final outcome	Desensitization	Acquired	Acquired	Acquired	Acquired	Acquired	Acquired	Not acquired	Acquired

In the LD group, 4 patients reached the dose that triggered the development of FPIES symptoms within 1 month after the start of CAFI and achieved desensitization within 3 months. In the ELD group, 3 patients reached the dose that triggered the development of FPIES symptoms at 5, 12, and 3 weeks after the start of CAFI, respectively, and achieved desensitization at 4 months.

∗ A dose of 1 g of egg yolk contains 165 mg of egg yolk protein, 1 g of boiled udon contains 26 mg of wheat protein, and 1 mL of soy milk contains 36 mg of soy protein.

† Dose at 3 months for the LD group and at 4 months for the ELD group.

In the ELD group, 3 patients reached the dose triggering FPIES development at 5, 12, and 3 weeks, respectively, after starting CAFI, and they achieved desensitization at 4 months (Table I). In contrast, a patient with egg yolk allergy did not continue consuming allergenic foods for even a week on account of abdominal symptoms.

In the LD and ELD groups, no patient presented with chronic diarrhea or weight loss that led to chronic FPIES during CAFI^1^ or relapsed after achieving desensitization and terminating CAFI.

Of the 21 patients, 4 (19.0%) had atypical FPIES, 3 developed FPIES symptoms at the initial OFC and ultimately received CAFI, and 2 achieved desensitization (see Table E1).

Of the 21 patients, 8 started CAFI and 7 (87.5%) achieved desensitization within 4 months after initiating CAFI. These results suggest the effectiveness of CAFI for acute FPIES.

The importance of the innate immune system’s response in the pathogenesis of FPIES has recently been reported.^4^^,^^5^ Monocyte activation was increased in the peripheral blood of patients who did not achieve tolerance with OFC.^4^ Therefore, CAFI for FPIES may improve the innate immune system’s response.^5^ The mechanism of allergenic food recognition in FPIES is expected to lead to the establishment of pathology-based therapies.

The presence of a threshold has been reported in patients with FPIES.^6^ Most patients with FPIES were reported to have a history of asymptomatic ingestion.^7^ These reports may support the significance of assessing thresholds and the usefulness of CAFI.

The initial dose of CAFI for assessing for acute FPIES was smaller than that required for assessing for IgE-mediated food allergy.^8^ This was due to acute FPIES patients experiencing severe symptoms even with reduced intake. The dose escalation rate was relatively high. Although further studies are needed, we believe that patients with acute FPIES require a smaller initial dose than do those with IgE-mediated food allergies but with a faster escalation rate.

Most patients with FPIES naturally achieve tolerance by age 3 years.^5^ The median age for starting for CAFI in this study was 33 months, which for many patients, is a difficult age at which to achieve tolerance; the exceptions were 3 patients who started CAFI before age 2 years. The age for starting CAFI was considered an issue for future study.

Atypical FPIES can be difficult to distinguish from IgE-mediated food allergies. Herein, atypical FPIES was defined as the occurrence of FPIES symptoms 3 hours after ingestion of allergenic food. IgE-mediated food allergy occurs up to 2 hours after allergenic food ingestion.^9^ Elucidating the pathogenesis and identifying biomarkers for acute FPIES may help clinicians differentiate the 2 conditions.^5^^,^^6^

This case series has some limitations, including its small sample size and insufficient immunologic evidence to explain the mechanism of CAFI’s therapeutic effect.

In conclusion, CAFI is a safe and efficient treatment for patients with a prolonged course of acute FPIES. Further studies with large sample sizes are needed to determine the optimal conditions for CAFI, including the initial dose, dose escalation rate, and starting age.
